# Complete Chloroplast Genome Sequence and Annotation of the *Saccharum* Hybrid Cultivar RB867515

**DOI:** 10.1128/genomeA.01157-16

**Published:** 2016-10-13

**Authors:** Pedro Marcus Pereira Vidigal, Alexandre Siqueira Guedes Coelho, Evandro Novaes, Márcio Henrique Pereira Barbosa, Luiz Alexandre Peternelli

**Affiliations:** a Programa de Melhoramento Genético da Cana-de-Açúcar (PMGCA-UFV), Universidade Federal de Viçosa, Minas Gerais, Brazil; b Núcleo de Análise de Biomoléculas (NuBioMol), Centro de Ciências Biológicas (CCB), Universidade Federal de Viçosa, Minas Gerais, Brazil; c Escola de Agronomia, Setor de Melhoramento de Plantas, Universidade Federal de Goiás, Goiás, Brazil; d Departamento de Fitotecnia, Universidade Federal de Viçosa, Minas Gerais, Brazil; e Departamento de Estatística, Universidade Federal de Viçosa, Minas Gerais, Brazil

## Abstract

Here, we present the chloroplast genome sequence of the *Saccharum* hybrid cultivar RB867515, the most planted sugarcane cultivar in Brazil.

## GENOME ANNOUNCEMENT

Sugarcane (*Saccharum* spp.) belongs to the *Poaceae* family. Due to its agricultural importance for sugar and biofuels industries, different efforts have been performed on sugarcane genomic research. Additionally, the chloroplast genomes of sugarcane cultivars SP80-3280 (1), NCo310 (2), and Q155 (3) have been determined and analyzed. These chloroplast genomes have a circular double-stranded DNA molecule containing a highly conserved sequence of 141.18 kb in length, with two inverted repeats (IR-A and IR-B) (1–3). Here, we present the complete chloroplast genome sequence of the *Saccharum* hybrid cultivar RB867515 (4). This sugarcane cultivar stands out due to its good productivity in sandy and low fertility soils, being the most planted sugarcane clone in Brazil in the past 5 years (5).

The total genomic DNA of RB867515 was extracted and used to produce eight genomic libraries, with insert sizes of 170, 500, and 800 nucleotides (nt). These libraries were sequenced with 2 × 100-bp paired-end reads using Illumina HiSeq 2000 by Beijing Genomics Institute (BGI, China). The reads were trimmed for quality (Q30 score) and filtered for length (90 nt) using Trimmomatic version 0.33 (6), producing a data set containing high-quality sequences with 90 to 100 nt. The chloroplast genome of RB867515 was assembled by a reference-guided approach using CLC Genomics Workbench version 6.5.1 (CLC bio). The genome sequences of SP80-3280, NCo310, and Q155 were aligned, and the consensus sequence was selected as a reference. The reads were mapped to the reference using global alignment (mismatch cost, 2; insertion cost, 3; deletion cost, 3; length fraction, 1.0; similarity fraction, 0.95) and 10.79 million sequences have been aligned with 7,675-fold coverage. Then, a contig sequence with 141,181 bp in length and a G+C content of 38.4% was obtained from mapped sequences by using the Extract Consensus Sequence tool. The contig sequence was opened to the same point of the SP80-3280 genome by precedent and was functionally annotated using DOGMA (7) and BLAST version 2.4.0 (8).

One hundred thirty-five genes were functionally annotated in the assembled chloroplast genome, 88 coding DNA sequences (CDSs), eight rRNA genes, and 39 tRNA genes. These genes cover 52.63% of chloroplast genome, excluding the intron sequences, and have a density of 0.95 genes/Kb. Twenty genes were annotated in both inverted repeats IR-A and IR-B, including four rRNA genes (*rrn4.5*, *rrn5*, *rrn16*, and *rrn23*), eight tRNA genes (*trnA*, *trnH*, 2 *trnI*, *trnL*, *trnN*, *trnR*, and *trnV*), and eight CDSs (*ndh*, *rpl2*, *rpl23*, *rps7*, *rps15*, *rps19*, *ycf2*, *ycf15*, and *ycf68*). The alignment of the sugarcane chloroplast genomes showed that sequences are highly conserved, with only 14 polymorphic sites identified, resulting in nucleotide identities greater than 99.99%. These polymorphic sites were previously described (3), being located at intergenic regions and at genes *atpA*, *psbC*, *rrn23*, *trnG*, *trnM*, and *trnS*. The RB867515 chloroplast genome is identical to the one of Q155 cultivar, differing from the NCo310 by five polymorphisms (four single-nucleotide polymorphisms [SNPs] and one indel) and from the SP80-3280 by eight polymorphisms (six SNPs and two indels). The genome sequence of RB867515 adds new information to the repertoire of chloroplast genomes of sugarcane, confirming that sequences of the sugarcane cultivars are highly conserved.

### Accession number(s).

The complete chloroplast genome sequence of sugarcane hybrid cultivar RB867515 is available in GenBank under the accession no. KX507245.
